# Redox regulation in regenerative medicine and tissue engineering: The paradox of oxygen

**DOI:** 10.1002/term.2730

**Published:** 2018-08-21

**Authors:** Mireille M.J.P.E. Sthijns, Clemens A. van Blitterswijk, Vanessa L.S. LaPointe

**Affiliations:** ^1^ Department of Complex Tissue Regeneration, MERLN Institute for Technology‐Inspired Regenerative Medicine Maastricht University Maastricht The Netherlands

**Keywords:** bone, heart, HIF, Nrf2, oxygen, pancreas, redox, redox regulating biomaterials

## Abstract

One of the biggest challenges in tissue engineering and regenerative medicine is to incorporate a functioning vasculature to overcome the consequences of a lack of oxygen and nutrients in the tissue construct. Otherwise, decreased oxygen tension leads to incomplete metabolism and the formation of the so‐called reactive oxygen species (ROS). Cells have many endogenous antioxidant systems to ensure a balance between ROS and antioxidants, but if this balance is disrupted by factors such as high levels of ROS due to long‐term hypoxia, there will be tissue damage and dysfunction. Current attempts to solve the oxygen problem in the field rarely take into account the importance of the redox balance and are instead centred on releasing or generating oxygen. The first problem with this approach is that although oxygen is necessary for life, it is paradoxically also a highly toxic molecule. Furthermore, although some oxygen‐generating biomaterials produce oxygen, they also generate hydrogen peroxide, a ROS, as an intermediate product. In this review, we discuss why it would be a superior strategy to supplement oxygen delivery with molecules to safeguard the important redox balance. Redox sensor proteins that can stimulate the anaerobic metabolism, angiogenesis, and enhancement of endogenous antioxidant systems are discussed as promising targets. We propose that redox regulating biomaterials have the potential to tackle some of the challenges related to angiogenesis and that the knowledge in this review will help scientists in tissue engineering and regenerative medicine realize this aim.

## 1 OXYGEN IN TISSUE ENGINEERING AND REGENERATIVE MEDICINE

**Figure 1:**
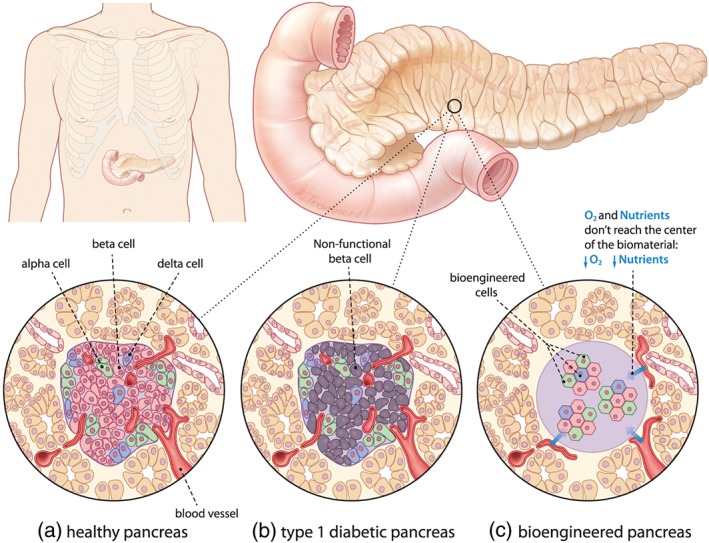
(a) Alpha and beta cells are organized in the highly vascularized islets of Langerhans in a healthy human pancreas, and their functions include maintaining balanced glucose levels. Alpha cells release glucagon in response to low blood glucose levels, ensuring sufficient energy supply in periods of starvation, whereas insulin is released by the beta cells directly after a meal, inducing storage of excess glucose. (b) In type 1 diabetes, the immune system attacks the beta cells, (c) and one option for a cure is a bioengineered pancreas of encapsulated islets of Langerhans that can be implanted at different sites (e.g., liver, peritoneum, or subcutaneous). However, because of reduced angiogenesis and subsequent low oxygen and nutrients levels, the transplanted cells may not function properly

One of the biggest challenges faced by tissue engineers is the engineering of a vasculature in their constructs (Stegen, van Gastel, et al., 2016). This vasculature is needed to deliver oxygen and other nutrients, and the lack thereof limits the size of tissue engineered constructs and is a major barrier to their successful function. As an example, one of the tissues that is very dependent on the delivery of oxygen is the (bioengineered) pancreas. In type 1 diabetes, the insulin‐producing beta cells are destroyed by the immune system. In order to cure this, one of the current therapies is the transplantation of de novo beta cells, but the success of this therapy is hindered by the lack of vascularization and accompanying low oxygen and nutrient levels (Figure 1). Indeed, oxygen is an essential metabolic requirement that needs to be delivered to cells, but it is also a molecule with a biological function. It is known to regulate stem cell fate (Simon & Keith, 2008; Tan & Suda, 2017; Ushio‐Fukai & Rehman, 2014; Veber, Dolivo, Rolle, & Dominko, 2017), with higher endogenous antioxidant levels seen in stem cells than in differentiated cells (Valle‐Prieto & Conget, 2010). When cells experience a lack of oxygen, or hypoxia, a rise in reactive oxygen species (ROS) is induced. A physiological rise in ROS leads to proliferation, whereas a larger increase in ROS induces differentiation (Tan & Suda, 2017; Ushio‐Fukai & Rehman, 2014). Apart from a direct effect, a lack of oxygen can influence the transcription factor hypoxia inducible factor (HIF) that affects Notch and Wnt/β‐catenin signalling to induce differentiation of endogenous neural stem and progenitor cells that are activated after stroke (Cunningham, Candelario, & Li, 2012).

Interestingly, in tissue engineering and regenerative medicine, oxygen is known as a life‐giving necessity. However, from a redox biology perspective, oxygen is considered an extremely toxic molecule (Sies, 2015). In this review, we explore this paradox, examine current approaches for delivering oxygen in tissue engineering, and propose that it is time for an alternative strategy inspired by redox biology.

## 2 THE IMPORTANCE OF THE REDOX BALANCE

**Figure 2:**
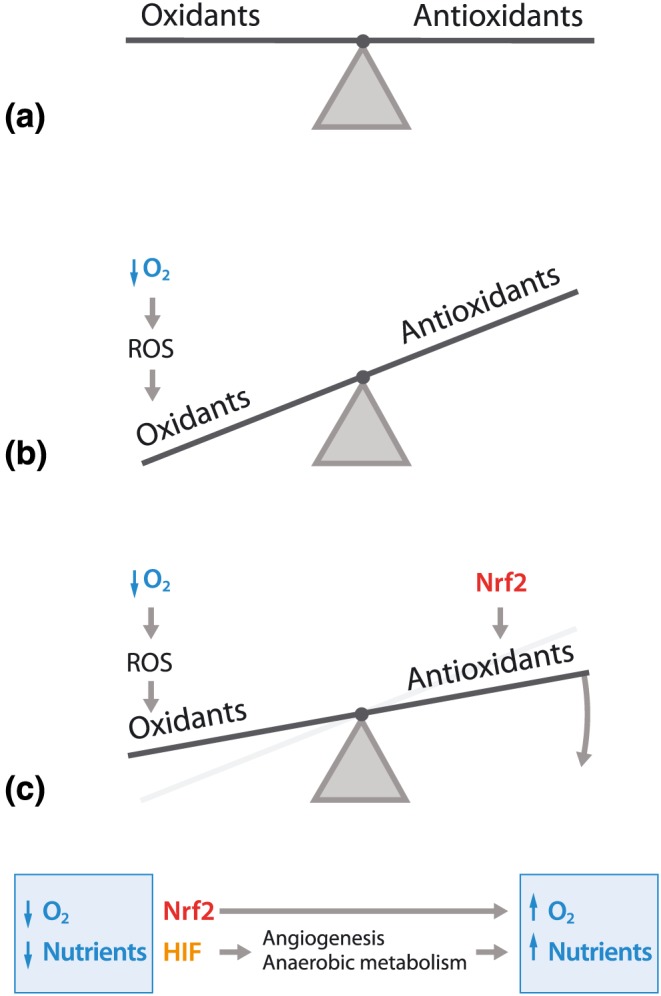
(a) In physiological conditions, cells have a tightly regulated and highly dynamic redox balance to maintain an equilibrium between oxidants and antioxidants. The cell is continuously exposed to different endogenous and exogenous oxidant and antioxidant challenges, but with endogenous oxidant generators and modulation of endogenous antioxidant systems, the cell is capable of maintaining the balance. (b) When the cell is exposed to more oxidants than the endogenous antioxidant systems can handle, this can disrupt the balance and induce a phenomenon called oxidative stress. This can happen, for example, when cells are exposed to an excess of oxygen, called hyperoxia. Hyperoxia dramatically increases the rate of aerobic metabolism, resulting in the generation of reactive oxygen species (ROS) due to incomplete metabolism, which in turn disrupts the redox balance and results in damage to cellular macromolecules, including DNA, lipids, and proteins. (c) The redox balance can be restored by targeting endogenous modulators of the endogenous antioxidant systems. For example, enhancement of the transcription factor Nrf2 increases (basal) levels of endogenous antioxidant systems (Sthijns et al., 2017), whereas increasing the transcription factor hypoxia inducible factor (HIF) induces anaerobic metabolism, stimulates angiogenesis, and increases glutaminase‐mediated glutathione synthesis, thereby enhancing endogenous antioxidant systems (Stegen, van Gastel, et al., 2016; Thirlwell, Schulz, Dibra, & Beck, 2011)

Functional tissues and organs maintain a tightly regulated balance between oxidants and antioxidants (Figure 2a). Oxidants are compounds that generate ROS such as radicals, whereas antioxidants scavenge radical species and prevent other compounds from being oxidized (Ursini, Maiorino, & Forman, 2016). The reactions induced by oxidants and antioxidants are collectively called redox reactions, or reduction and oxidation reactions, respectively.

Cells can experience higher concentrations of oxidants due to endogenous factors such as enhanced aerobic metabolism, or exogenous factors such as radiation (Figure 2b). Fortunately, every cell is equipped with multiple endogenous antioxidant systems including the glutathione (GSH) system, thioredoxin system, different vitamins, and protective enzymes such as catalase or superoxide dismutase that can be upregulated to restore the redox balance on demand. In this regulation, different redox regulated transcription factors are involved, for example, nuclear factor (erythroid‐derived 2)‐like 2 (Nrf2) or HIF (Figure 2c). These endogenous antioxidant systems are compartmentalized. For example, GSH and superoxide dismutase are present at high levels in mitochondria, where aerobic metabolism takes place, whereas vitamin E can mainly be found in the plasma membrane.

During oxidative stress, oxidants such as ROS are generated in excess relative to endogenous antioxidant levels, and the balance cannot be maintained (Sies, 2015). Although a short‐term and relatively small increase in ROS is necessary for the redox signalling that is important in processes such as inflammation (NADPH oxidases) or angiogenesis (HIF‐regulated; Sthijns, Weseler, Bast, & Haenen, 2016), a long‐term and relatively large rise in ROS induces damage to essential cellular macromolecules, DNA, proteins, or lipids that may ultimately lead to the development of diseases such as diabetes (Gough & Cotter, 2011). For example, the onset of both type 1 and type 2 diabetes appear to be partly caused by beta cell dysfunction following oxidative stress (Fridlyand & Philipson, 2004). Indeed, beta cells are very sensitive to oxidative stress because they contain lower endogenous antioxidant levels than many other cell types (Robertson, Harmon, Tran, Tanaka, & Takahashi, 2003). And the high blood glucose levels characteristic in diabetes lead to increased metabolism that induces the formation of oxidative stress by, for example, reducing the NADPH that is necessary for maintaining the level of the antioxidant GSH (the polyol pathway; Brownlee, 2001) or by overloading the electron transport chain thereby inducing the formation of ROS. Subsequently, the formed ROS contribute to the pathology of diabetes in multiple ways. First of all, the expression of insulin mRNA and other genes important in its regulation (e.g., *GLUT‐4*) are decreased, as is the binding of the transcription factor PDX1 to its promotor site (Kajimoto & Kaneto, 2004; Rains & Jain, 2011). On the protein level, oxidation of Ser/Thr protein kinases leads to the phosphorylation and activation of the insulin receptor substrate‐1 that inhibits downstream phosphatidyl‐inositol‐3‐kinase activation, thereby impairing glucose transport, glycogen formation, and gluconeogenesis and further contributing to insulin resistance in diabetes (Tiganis, 2011). And finally, when focusing on lipids, lipid peroxidation products are mediators of the immune system involved in the degradation of pancreatic beta cells, which can further deteriorate the status of the patient (Tangvarasittichai, 2015). Beyond diabetes, oxidation of cellular DNA, proteins, and lipids can contribute to a wide range of pathologies, including cardiovascular or neurodegenerative diseases (Maher, 2017; Ooi, Goh, & Yap, 2017).

## 3 OXYGEN‐RELEASING AND OXYGEN‐GENERATING BIOMATERIALS

Knowing its importance in biology, many biomaterials have been designed to either release or generate oxygen (Farris, Rindone, & Grayson, 2016; Gholipourmalekabadi, Zhao, Harrison, Mozafari, & Seifalian, 2016; Harrison, Eberli, Lee, Atala, & Yoo, 2007; Ma et al., 2016). The so‐called oxygen‐releasing biomaterials comprise the oxygen carriers, including perfluorocarbons, silicone oils, or crosslinked haemoglobin (Kimelman‐Bleich et al., 2009; Radisic et al., 2006). The most common oxygen‐generating biomaterials contain peroxides, sodium percarbonate, calcium oxide (Coronel, Geusz, & Stabler, 2017), cerium oxide (Mahapatra et al., 2017; Marino et al., 2017), magnesium oxide (Roh et al., 2017), or fluorinated materials. However, the success of these materials is limited by a major biological challenge because hyperoxia induces the formation of ROS by dysregulating the redox balance, and this can have dire effects on cell survival and function. Further complications are present when the material contains peroxides, as these also generate hydrogen peroxide (H_2_O_2_) upon contacting water (Farris et al., 2016). H_2_O_2_ is one of the ROS that damages essential cellular macromolecules, and in response, lipid peroxidation is induced (D'Agostino, Olson, & Dean, 2009), proteins are oxidized (Awasthi, Gyurasics, Knight, Welty, & Smith, 1998), and DNA strand breaks and associated mutations are provoked (Cacciuttolo, Trinh, Lumpkin, & Rao, 1993), potentially leading to cellular dysfunction and death (Wijeratne, Cuppett, & Schlegel, 2005). It should be clear that the use of oxygen‐releasing or oxygen‐generating biomaterials should be considered with caution, as many of the intended positive effects of supplying oxygen may be accompanied by devastating effects of oxidative stress.

## 4 BIOMATERIALS MODULATING THE ENDOGENOUS ANTIOXIDANT SYSTEMS

Knowing that cells need oxygen to survive, but that supplying oxygen carries a sizeable risk of disturbing the redox balance and leading to oxidative stress, some scientists have engineered materials that better reflect an understanding of the complicated nature of redox biology. One promising approach is to use drug delivery strategies that directly incorporate antioxidant enzymes such as catalase in biomaterials in order to maintain the redox balance even in the presence of oxygen (Gholipourmalekabadi et al., 2016; Luo, O'Reilly, Thorpe, Buckley, & Kelly, 2016). In this case, an endogenous antioxidant enzyme present in every living cell is added to the oxygen‐generating biomaterials to scavenge the released H_2_O_2_ intermediate product and convert it to water and oxygen. The challenge with this approach is that antioxidants, like other drugs, have a narrow effective range. Indeed, too much antioxidant can also be toxic or is associated with diseases related to decreased proliferation or immunosuppression (Bast & Haenen, 2013; Galadari, Rahman, Pallichankandy, & Thayyullathil, 2017; Schieber & Chandel, 2014). Nonetheless, with careful modulation of the release profile, it should be possible for a biomaterial to help maintain the redox balance.

**Figure 3:**
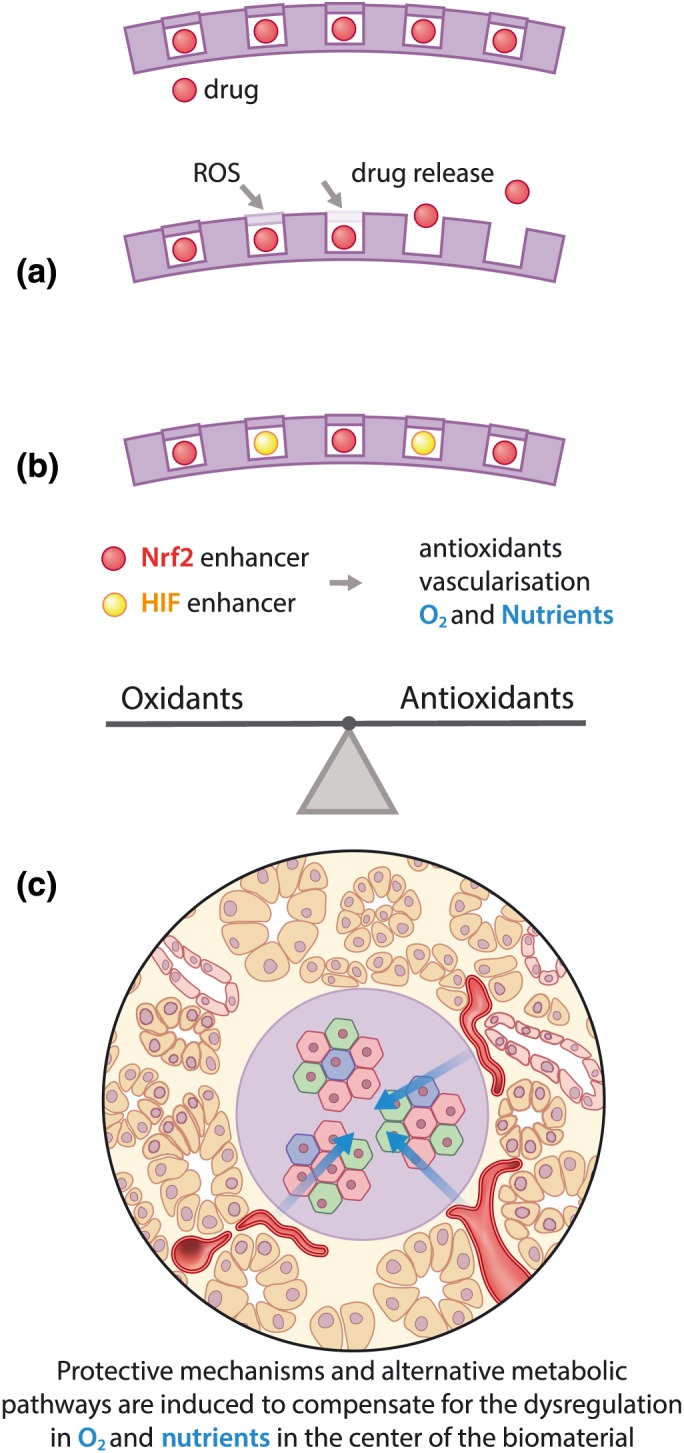
(a) A superior strategy for redox modulation could be to design a biomaterial that releases a drug in presence of excess reactive oxygen species (ROS) or oxidative stress. (b) The molecular targets are the redox sensor transcription factors Nrf2 and hypoxia inducible factor (HIF). By modulating Nrf2, the regulation of endogenous antioxidant systems (Sthijns et al., 2017) is induced, resulting in restoration of the redox balance. HIF activation increases anaerobic metabolism, stimulates angiogenesis, and increases glutaminase‐mediated glutathione synthesis, thereby enhancing endogenous antioxidant systems to recover the redox balance (Stegen, van Gastel, et al., 2016; Thirlwell et al., 2011). (c) Introducing Nrf2 and HIF enhancers in a biomaterial for pancreatic islets could prevent damage from oxidative stress, induce alternative metabolic pathways to ensure a sufficient energy supply, and enhance angiogenesis

A more elegant approach could be to supplement oxygen delivery with the modulation of endogenous antioxidant systems by designing a scaffold that is responsive to ROS levels (Martin et al., 2014; Tang et al., 2015; Figure 3a). For example, this could be done by using poly(thioketal) urethane or poly‐(1,4‐phenyleneacetone dimethylene thioketal) in tissue engineered scaffolds or nanoparticles for drug delivery in the presence of ROS. A biomaterial could be designed to only release oxygen in hypoxic conditions or to release oxidants or antioxidants in response to the local ROS levels.

## 5 MOLECULAR TARGETS FOR TISSUE ENGINEERS

Because simply supplementing oxygen or introducing antioxidants into a biomaterial can still interfere with the redox balance and lead to undesirable outcomes, it is important for scientists in tissue engineering and regenerative medicine to have some knowledge about the essential proteins in redox regulation, so they can monitor or even modulate the cellular response. Redox sensor proteins are involved in detecting the dynamic balance between oxidants and antioxidants in the cell and driving the intracellular feedback mechanisms to increase the synthesis of important endogenous antioxidant systems or to regulate intracellular ROS signalling (Brigelius‐Flohe & Flohe, 2011). High‐throughput screening of cellular redox sensors using modern redox proteomics revealed that redox‐related transcription factors *NRF2* and *HIF* are promising targets for modulation, especially on the antioxidant side of the balance (Jiang, Wang, Nice, Zhang, & Huang, 2015; Figure 3b).

For example, Nrf2 is a redox sensor protein that works to upregulate endogenous antioxidant systems, but it also takes into account the local endogenous antioxidant levels and ROS. The mechanism for upregulating endogenous antioxidant systems is through Keap1, an Nrf2 inhibitor that normally directs Nrf2 for proteasomal degradation. Upon encountering ROS, however, newly synthesized Nrf2 is directed to the nucleus and functions as a transcription factor to upregulate the expression of antioxidant genes (e.g., *GCLC*, the rate‐limiting enzyme for GSH synthesis, which is one of the major endogenous antioxidants). This pathway is also an example of the precise balance between oxidants and antioxidants because Nrf2 has three mechanisms through which it can respond to endogenous antioxidant levels. First, *GCLC* expression is not continuously upregulated. Nrf2 induces a negative feedback loop, thereby decreasing the expression of *GCLC* and the formation of endogenous GSH (Kaspar & Jaiswal, 2010). Second, GSH depletion itself (when ROS is absent) also induces Nrf2 activation (Chia et al., 2010). Finally, the formed protein GCL has a catalytic subunit that only functions when GSH levels are low, meaning that GSH synthesis by GCL is only increased when low levels of GSH are present (Huang, Chang, Anderson, & Meister, 1993; Rahman, Bel, Mulier, Donaldson, & MacNee, 1998; Rahman & MacNee, 1999; Sthijns et al., 2016; Tian, Shi, & Forman, 1997). Therefore, the added benefit form targeting the Nrf2 pathway for modulation is that it naturally keeps the antioxidant and oxidant balance into account.

Another strategy is to target the oxidant side of the balance by regulating the activity of the most important endogenous generator of ROS, NADPH oxidase (Panday, Sahoo, Osorio, & Batra, 2015). Importantly, NADPH oxidases also take into account the redox balance, because they contain a sensor for the amount of endogenous H_2_O_2_ present and regulate their activity based on that (Nisimoto, Ogawa, Kadokawa, & Qiao, 2018).

Interestingly for tissue engineering, these proteins are not just central to redox signalling but are also potential targets for modulation by a biomaterial or other strategies (Figure 3c). Indeed, many different release strategies already exist, such as the entrapment and controlled release of bioactive factors from engineered matrices, so modulation of redox factors is a reasonable reality (van Blitterswijk et al., 2008). And the evidence for the need of such a strategy is seen in the involvement of HIF, Nrf2, and NADPH oxidase in the regeneration of both highly oxygen‐demanding tissues (e.g., brain/neurons, heart, and pancreas) and low oxygen tissues (e.g., bone and cartilage), which is explored in the remainder of this section.

### 5.1 HIF pathway

Apart from its role as an important redox sensor protein involved in stimulating anaerobic metabolism, angiogenesis, and enhancing endogenous antioxidant systems (Stegen, van Gastel, et al., 2016; Thirlwell et al., 2011), HIF is also an important regulator in the regeneration of different tissues including neurons, heart, bone, cartilage, and pancreas. In neurons, low oxygen tension induces ROS, which is a signal for neurogenesis (Hameed et al., 2015; Zeng, Kamei, Wang, & Tsai, 2016), and HIF1α signalling enhances axon regeneration (Alam et al., 2016; Cho et al., 2015), which is also linked to ROS levels (Quinta et al., 2016). In the heart, *Hif1α* is essential for cardiomyogenesis (Kudova et al., 2016), and the hypoxia experienced after myocardial infarction is known to enhance the systolic function of the left ventricle and prevent fibrosis in mice (Nakada et al., 2017). On the other hand, high oxygen levels induce cardiomyocyte cell cycle arrest but enhance cardiomyocyte function (Carrier et al., 2002; Puente et al., 2014). During bone fracture repair, *Hif* upregulation is seen in rats (Komatsu & Hadjiargyrou, 2004), and HIF1α is known to be essential in both bone and cartilage repair (Jiang et al., 2016; Kanichai, Ferguson, Prendergast, & Campbell, 2008; Stegen, Deprez, et al., 2016; Stegen, van Gastel, et al., 2016; Wan et al., 2008; Zou et al., 2012).

Taken together, it is clear that HIF is not only important but also an essential player in the regeneration of various damaged tissues, which underlines the promise of an approach using HIF inducers in biomaterials. For example, the addition of deferoxamine, a HIF inducer, into a 3D‐bioresorbable bone graft substitute increased bone formation (Cahill, Choudhury, & Riley, 2017; Drager et al., 2017). Furthermore, deferoxamine also has been shown to improve the success of human islet transplantation, a therapy used in regenerative medicine to replace the damaged beta cells in the pancreas, thereby motivating its application in other tissues (Stokes et al., 2013).

### 5.2 NADPH oxidase

Another important redox modulator that has not yet been a major target in tissue engineering is NADPH oxidase. NADPH oxidase induces ROS formation (superoxide), which is necessary for redox signalling. In terms of applications in tissue regeneration, NADPH oxidase also plays an important role in the regeneration of damaged tissues. For example, NADPH oxidase‐dependent ROS formation stimulates the differentiation of murine pancreatic progenitor cells into endocrine cells and thereby enhances pancreatic beta cell regeneration (Liang et al., 2016) while inhibiting proliferation (Wang & Wang, 2017). In addition, NADPH oxidase‐induced ROS formation also contributes to the differentiation of cardiomyocytes from embryonic stem cells (Buggisch et al., 2007). The NAPDH oxidase inhibitors apocynin and diphenyleneiodonium (Altenhofer, Radermacher, Kleikers, Wingler, & Schmidt, 2015; Drummond, Selemidis, Griendling, & Sobey, 2011), small molecule pharmacological inhibitors of the enzyme, could be easily incorporated in a redox regulating biomaterial to further leverage their positive effects for tissue engineering and regenerative medicine.

### 5.3 Nrf2 pathway

Nrf2 has likewise not yet been a major target in tissue engineering approaches. Nrf2 enhances the expression of endogenous antioxidant systems, and multiple studies underline its essential role in regulating the regeneration of pancreatic, cardiac, and bone tissue. Specifically, deletion of the transcription factor *Nrf2* in diabetic mice exacerbates hyperglycaemia (Aleksunes, Reisman, Yeager, Goedken, & Klaassen, 2010), whereas enhancing Nrf2 improves diabetic wound healing (Soares et al., 2016). The loss of Nrf2‐downstream target NAD(P)H:quinone oxidoreductase 1 induced beta cell destruction and confirms the essential modulating role of Nrf2 in beta cell function (Yeo et al., 2013). In the heart, Nrf2 prevents pathological hypertension‐induced cardiac remodelling and concomitant heart failure (Zhou, Sun, Zhang, & Zheng, 2014) and is essential for cardiac repair after injury (Tao et al., 2016). Finally, *Nrf2* upregulation is detected during bone fracture repair in rats (Komatsu & Hadjiargyrou, 2004), and inhibition of thioredoxin, one of the essential endogenous antioxidant systems mediated by Nrf2, reduced fracture healing (Muinos‐Lopez et al., 2016). All in all, targeting Nrf2 is a promising strategy to enhance the regenerative capacity of numerous tissues. One approach could be to supplement existing biomaterials with Nrf2 small molecular modulators such as auranofin, sulforaphane, or *tert‐*butylhydroquinone (Ma, 2013) to enhance endogenous antioxidant systems, restore the redox balance, and improve the survival and function of the engineered tissue.

## 6 PERSPECTIVE ON NEW STRATEGIES

Compared with current approaches for oxygen delivery, the advantage of modulating endogenous antioxidant systems is that the problems with oxygen toxicity are avoided. The endogenous redox balance must be considered, as it is the redox‐regulating systems themselves that are modulated in cells and not the antioxidant systems or ROS levels alone. Furthermore, because the mechanisms related to the redox balance are present in all tissues, their modulation is widely applicable, unlike, for example, tissue‐specific growth factors. Finally, scientists can benefit from the fact that redox modulation is essential for both regeneration and angiogenesis, which suggests great promise for overcoming many of the challenges in tissue engineering and regenerative medicine.

## CONFLICTS OF INTEREST

The manuscript has not been published and is not under consideration for publication elsewhere. We have no conflicts of interest to disclose. All the work conducted in this manuscript has been conducted under internationally accepted ethical standards.
